# Shortcut learning in medical AI hinders generalization: method for estimating AI model generalization without external data

**DOI:** 10.1038/s41746-024-01118-4

**Published:** 2024-05-14

**Authors:** Cathy Ong Ly, Balagopal Unnikrishnan, Tony Tadic, Tirth Patel, Joe Duhamel, Sonja Kandel, Yasbanoo Moayedi, Michael Brudno, Andrew Hope, Heather Ross, Chris McIntosh

**Affiliations:** 1 Peter Munk Cardiac Centre and Ted Rogers Centre for Heart Research, University Health Network, Toronto, ON Canada; 2 Department of Medical Biophysics, University of Toronto, Toronto, ON Canada; 3 Toronto General Hospital Research Institute, University Health Network, Toronto, ON Canada; 4 Department of Computer Science, University of Toronto, Toronto, ON Canada; 5 Joint Department of Medical Imaging, University Health Network, Toronto, ON Canada; 6 Vector Institute, Toronto, ON Canada; 7 Radiation Medicine Program, Princess Margaret Cancer Centre, University Health Network, Toronto, ON Canada; 8 Department of Radiation Oncology, University of Toronto, Toronto, ON Canada; 9 Princess Margaret Cancer Centre, University Health Network, Toronto, ON Canada; 10 Department of Medical Imaging, University of Toronto, Toronto, ON Canada

**Keywords:** Machine learning, Data acquisition

## Abstract

Healthcare datasets are becoming larger and more complex, necessitating the development of accurate and generalizable AI models for medical applications. Unstructured datasets, including medical imaging, electrocardiograms, and natural language data, are gaining attention with advancements in deep convolutional neural networks and large language models. However, estimating the generalizability of these models to new healthcare settings without extensive validation on external data remains challenging. In experiments across 13 datasets including X-rays, CTs, ECGs, clinical discharge summaries, and lung auscultation data, our results demonstrate that model performance is frequently overestimated by up to 20% on average due to shortcut learning of hidden data acquisition biases (DAB). Shortcut learning refers to a phenomenon in which an AI model learns to solve a task based on spurious correlations present in the data as opposed to features directly related to the task itself. We propose an open source, bias-corrected external accuracy estimate, *P*_*Est*_, that better estimates external accuracy to within 4% on average by measuring and calibrating for DAB-induced shortcut learning.

## Introduction

Through the advent of deep learning (DL), AI has made dramatic leaps in accuracy for many challenging healthcare problems, with multiple models stepping closer to wide-scale clinical deployment. However, generalization to novel data remains a significant barrier, with multiple studies having demonstrated that accuracy often decreases by large margins in novel deployment^1–5^. At the same time, without access to external datasets, identifying what the external accuracy of a model will be or the main drivers of reductions in accuracy (e.g., data drift or overfitting) remains challenging.

A frequently proposed solution to increase model accuracy and generalizability is through extremely large datasets. Labeled medical datasets of over 10k patients are sought with the assumption that a large enough dataset will capture the necessary variability in both training and validation to be representative of future performance. Although these datasets could be collected prospectively and purposefully for the purpose of training an AI model, most patients receive standard of care instead of participating in clinical trials. Thus, most large datasets are collected through what we call *passive* collection.

In *passive* collection, the data is gathered through routine clinical care across as wide a net as possible. The advantage is that large-scale data collection becomes fast, scalable, and straightforward. Instead of hundreds of patients, some datasets have grown to reach tens or even hundreds of thousands of patients through this methodology. However, we believe that there is a hidden problem with this method of dataset creation that, combined with the limitations of modern DL models, is a root cause of model generalization challenges in healthcare.

We hypothesize that a significant cause of the lack of generalizable AI models stems from the shortcut learning of an under-reported data acquisition bias (DAB) that naturally occurs during passive data collection, and if we could estimate the impact that bias has on a model, we could use it to better estimate model accuracy on external data and measure the quality of a dataset for model building. Different hospital wards encounter varying proportions of medical conditions and use different acquisition conditions, hardware, or settings. These medical condition-specific acquisition parameters, although not perceptible to the human eye, are detectable by machine learning (ML) algorithms. When data is collected through routine care, these imperceptible signal changes are heavily correlated with disease label, and get leveraged by the model as surrogates. Spurious features manifest in AI models through a process termed shortcut learning, wherein a model learns a readily AI-visible indicator of a sample class instead of a more subtle one that ultimately generalizes better to novel data^6^. For example, in medical imaging, the contrast of the image can be influenced by medical imaging acquisition parameters. The model may then use the contrast as a surrogate for predictions or decisions as a shortcut. Acquisition parameters are not standardized and may vary from institution to institution or within an institution over time, and thus, if learned as shortcuts, may negatively impact model generalizability. These DABs are analogous to batch effects which arise from variation in data collection due to non-biological factors^7,8^.

Batch effects are a well-studied issue in gene expression experiments where the processing date and parameters of batches may play a larger role in differentiating between disease groups than the underlying gene expressions^7,8^. While methods exist to mitigate batch effects in molecular biology, they remain largely unexplored in the broader field of AI. Particularly in their application to unstructured clinical data (images, electrocardiograms, medical charts, etc., as opposed to tabularized data)^7–10^. Previous studies have pooled MRI datasets from multiple centers to build larger datasets for their ML algorithms demonstrating that acquisition parameters impact results in the context of traditional analysis methods^11–13^ and two studies have examined batch effects in DL limited to histopathology data^14,15^. A study using DL methods to detect COVID-19 in chest radiographs found that shortcut learning of confounding data acquisition factors impacted model performance when testing on external datasets and identified that collecting less confounded data was the most reliable way of alleviating the issue^5^. Other traditional accuracy estimation approaches include cross-validation, which estimates the variance in performance across different samplings (folds) of the population captured by the available data, but does not address DAB-induced performance degradation on unseen external datasets as DAB will be equally distributed across the available training and test folds.

The field lacks a comprehensive study investigating the impact of DAB on DL models within the broader context of unstructured clinical data. Additionally, as external datasets are often unavailable, a method is needed to evaluate how DAB may affect model performance on said datasets without requiring access to them. Furthermore, it is insufficient to simply quantify the batch effects within a dataset irrespective of a model, as a particular model architecture might be immune to a particular batch effect, but not others. Therefore, a method is needed to quantify the degree of DAB-induced shortcut learning (DABIS) under any given DL model. To address these gaps, we propose a method to improve AI validation in medical classification tasks on unstructured data without the need for an external validation dataset or without explicit prior knowledge of bias in a dataset, and apply the method to a wide array of data types.

Our study aims to elucidate a potential cause and quantify the significant reduction in accuracy seen when medical AI models are externally validated. We propose a method to better estimate the external accuracy of AI models in healthcare on unstructured data through a straightforward assumption that can be made without explicit prior knowledge of the DAB in a dataset or an external validation dataset (i.e. the most general case).

**Fig. 1:**
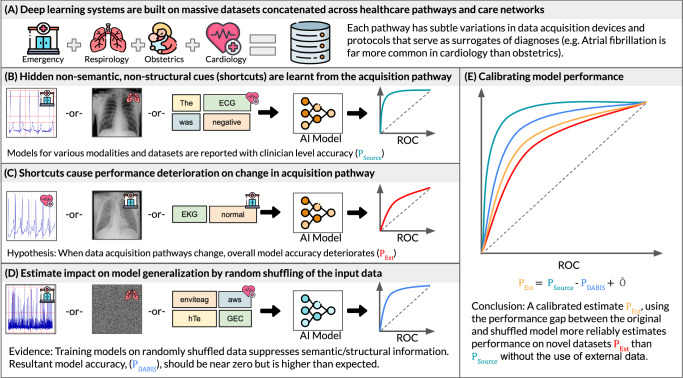
Data acquisition induced bias in AI systems in healthcare. Data collection and existing/proposed AI development and validation workflows in healthcare. **A** Datasets for deep learning are commonly collected across different healthcare pathways (emergency, respirology, obstetrics, cardiology, etc.) or care networks, and each pathway or hospital uses slightly different data acquisition hardware and protocols. **B** Models are reported to have clinical-level accuracy, but they do so by learning hidden non-semantic and non-structural cues from the acquisition pathway **C** The performance does not generalize to other hospitals with different data acquisition pathways. We hypothesize that this is because the models have learned to use subtle data acquisition features as surrogates of diagnoses. **D** We randomly shuffle the data within each patient sample to suppress structural and semantic information. If datasets were unbiased, resultant models would have near zero accuracy, but their performances are higher than anticipated. **E** In our proposed workflow, data acquisition bias induced shortcut learning (DABIS) estimated using signal shuffling enables the reporting of calibrated accuracy measures that are more reflective of external validation accuracy.

Our approach has two parts: first, to estimate the degree of DABIS in a dataset with a specific model; and then to calibrate the test set accuracy by that amount. To estimate the DABIS, we assume that reliable medical signals (i.e. those that will externally generalize) are based on spatial or temporal relationships of the data within a sample (i.e. the structural and semantic features) instead of the frequency of individual pixel brightness levels, or letters. Consider the examples in Fig. 1B. The ECG, X-ray, or sentence are individually readily understood by experts based on the local order of their data points (i.e. the structure of the data), and are used to train AI models. The same experts readily understand slightly different data from another hospital as the nature of the structure and semantic features are unchanged (Panel C); however, the AI models lose significant accuracy. The datasets are concatenations across different hospital acquisition pathways, with higher ratios of sick patients naturally falling under a particular X-ray scanner, for example. Compare panels B and D, the same samples have been randomly shuffled, altering the data’s structural features and changing its semantic meaning. An expert can no longer interpret the data, but an AI model trained on the shuffled data has high accuracy implying that it is learning something different than the experts. It is not learning structural and semantic features relevant to clinical diagnoses/interpretation, but instead learning other features from the data’s histogram such as first-order statistical and frequency features. We argue that structural and semantic features are more reproducible across different hospital data pathways (e.g. different scanners), and thus better represent model performance across different hospitals, or over time within a hospital. For example, the ratio of dark to bright pixels in an X-ray should not be used to make a diagnosis since it changes based on the field of view or brand of scanner, but rather the model should rely instead on the structure of the patient anatomy within the image. Based on this assumption, we hypothesized that randomly shuffling spatial and temporal components of the data will remove the most reliable features (structural and semantic), forcing the AI model to instead focus on potential DAB, and giving us an estimate of the magnitude of their impact on the model (Panel D). We further use the shuffled model accuracy to better estimate accuracy for external datasets, when they are unavailable, compared to current reporting methods through a calibration step (Panel E). For a broad study of this phenomenon, we used a wide array of medical data from multiple clinically diagnostic modalities and hospital networks.

## Results

### Dataset characteristics

We assembled data from 207,487 patients across five modalities of unstructured data (X-Ray, computed tomography (CT), electrocardiogram (ECG), lung auscultation, and clinical discharge summaries from electronic health record (EHR) and 13 unique datasets (Table 1). We trained and externally validated our models on nine public and four internal datasets, as described in (Table 1). All internal datasets were collected with institutional approvals. To evaluate trained models on external validation datasets, we had to ensure the training datasets and external datasets had the same target labels and the same modality from a different site. For datasets that were multiclass, we narrowed down the class labels to those that intersected between the source and external validation datasets (Table 1). In the case of the ECG datasets we further categorized the external validation dataset labels into the super-classes of the source model dataset. The individual dataset details are presented in the Methods section. Additional experiments were also conducted with combinations of datasets denoted as A + B.

**Table 1:** Dataset Characteristics

Database	#Patients	#Samples	Modality	#Classes	Classes (Evaluated classes are in bold)
MIMIC-CXR	65379	377095	X-Ray	14	No Finding, Enlarged Cardiomediastinum, **Cardiomegaly,**
CXP	64540	223414	X-Ray		Lung Opacity, Lung Lesion, **Edema,** **Consolidation,**
MIMIC-CXR+CXP	98964	300000	X-Ray		Pneumonia, **Atelectasis**, Pneumothorax, **Pleural Effusion,**
MIMIC-CXR+NIH	62545	172562	X-Ray		Pleural Other, Fracture, Support Devices
NIH	30763	111788	X-Ray	15	**Atelectasis,** **Cardiomegaly,** **Consolidation,** **Edema,** **Pleural Effusion**, Emphysema, Fibrosis, Hernia, Infiltration, Mass, No Finding, Nodule, Pleural Thickening, Pneumonia, Pneumothorax
COVID-Kaggle	10192	21165	X-Ray	4	**COVID Positive,** **Normal**, Lung Opacity, Viral Pneumonia
COVID-Internal	2962	10731	X-Ray	2	**COVID Positive,** **Normal**
ILD-Diag	3182	4394	CT	2	**ILD,** **No ILD**
ILD-Plan	503	503	CT	2	**ILD,** **No ILD**
PTB-XL ECG	18885	21837	ECG	5	**Conduction Disturbance,** **Hypertrophy**, Myocardial Infarction, ST/T Change, Normal ECG
LUDB	157	157	ECG	2	**Conduction Disturbance,** **Hypertrophy**
ICBHI	97	866	Auscultation	2	**Normal,** **Abnormal**
JUST	243	243	Auscultation	2	**Normal,** **Abnormal**
MIMIC-III	4292	26244	EHR	2	**Readmission,** **no readmission**
EHR-Int	6292	7263	EHR	2	**Readmission,** **no readmission**
**Total unique:**	207487	805700			

Details of datasets sizes and label classes used in experiments. All datasets were split by 80/20 with the exception of external validation only datasets: COVID-Internal, LUDB, and JUST. All dataset splits occurred at the patient level. **Public Datasets**: MIMIC-CXR (Medical Information Mart for Intensive Care-Chest X-ray), MIMIC-III, Stanford CheXpert Chest X-rays (CXP), COVID-Kaggle, National Institute of Health (NIH) X-ray dataset, Physikalisch-Technische Bundesanstalt-XL ECG (PTB-XL), Lobachevsky University Electrocardiography Database (LUDB), International Conference on Biomedical Health Informatics (ICBHI 2017), Jordan University of Science and Technology Faculty of Computer and Information Technology & King Abdullah University Hospital (JUST). **Internal Datasets**: Interstitial Lung Disease CT (ILD-Diag, and ILD-Plan), COVID Internal(COVID-Int), Electronic Health Record (EHR-Int) clinical discharge summaries.

### Baseline models

To assess the influence of DABIS on model performance, we trained a common practice representative DL model for each task with convolutional neural network (CNN) models using either a DenseNet^16^ or an adaption of VGGNet^17^ for spatial or temporal data, and a large language model (LLM) for the natural language data in clinical discharge summaries. Specifically, we utilized BERT^18^, a bidirectional encoder pretrained with masked language modeling, and fine-tuned it for clinical notes and discharge summaries following Huang et al.^19^ and Jiang et al.^20^. Models were designed to represent commonly used choices, not be custom designed for each problem. Each model was trained independently for the given dataset in the normal fully supervised manner (details in Methods). We call these the ‘source’ datasets.

### Estimation of bias from training data

The first step is to estimate the potential dataset bias that is learnable by the model by using one or more data transforms to reduce meaningful signals while preserving bias. We propose a highly general transform by randomly shuffling all elements within each data sample along its structured axes, e.g., spatial, temporal, order of words, and order of letters within a word. Data shuffling in this manner eliminates all structural and semantic features, i.e. texture, or structure in an X-ray, or QRS complexes on an ECG, or meaning of a sentence, and renders the data uninterpretable to human experts. Examples of an X-ray, an ECG, and a simple sentence are shown in Fig. 1B, with corresponding shuffled samples in panel D. We hypothesize that if a dataset is free of DAB with respect to a particular model then there will be no hidden features left for a model trained on shuffled data to learn, and its accuracy will be that of a purely random model. Conversely if there are hidden DAB, the resultant trained model will have greater than random accuracy. The only preserved information after shuffling are data histograms, which we demonstrate as being unconsciously, at least to the researchers, over-emphasized by the models. Random shuffling is repeated for each iteration (epoch) through the entire dataset. We do not shuffle across data channels, e.g. leads of an ECG, as we consider them to have independent biases. A pseudocode algorithm is presented in Alg. 1. The accuracy of these models when trained and evaluated on shuffled data forms our DABIS estimate, *P*_*DABIS*_.

Next, the DABIS estimate can be used to predict the model accuracy on an unseen external dataset by calibrating it as follows:

1 $${P}_{Est}={P}_{Source}-{P}_{DABIS}+\bar{O}$$

where *P*_*Est*_ is our estimated performance on external data, *P*_*Source*_ is the performance of a model trained in the normal manner on the dataset, *P*_*DABIS*_ is the performance of the model trained on shuffled data, and $\bar{O}$ is the accuracy of a purely random model. For our experiments we used the well-reported area under the receiver operating curve (AUROC). The AUROC treats all classes as equally probable and is thus invariant to the underlying class distribution enabling fair performance comparisons across diverse datasets with different target populations and diseases. For multi-class problems, the average AUROC is reported. For AUROC we set $\bar{O}=0.5$, indicating a random model. During the bias estimation and correction, we similarly calibrate the ROC curves themselves. We present the pseudocode of the entire process in Alg. 2.

**Fig. 2:**
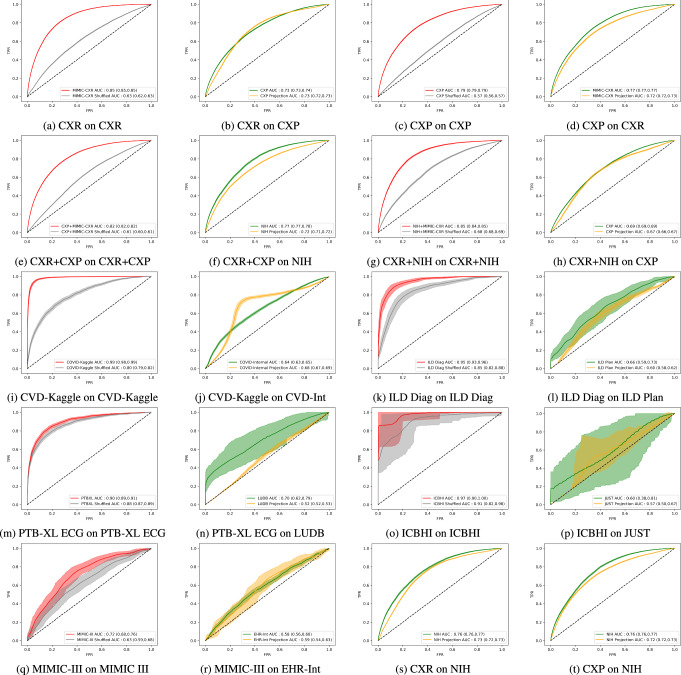
Comparison of source data model receiver operator curves (ROC), estimated external validation ROC, and observed external validation ROC on 13 datasets and 5 modalities. Model receiver operator characteristic curves on source (**a**, **c**, **e**, **g**, **i**, **k**, **m**, **o**, **q**) and external validation datasets (**b**, **d**, **f**, **h**, **j**, **l**, **n**, **p**, **r**, **s**, **t**). Source dataset figures include the corresponding DABIS estimate (gray) and the external dataset figures include our estimated curves (yellow). Shaded regions depict the 95% confidence interval. Notice that the ROC curves on the external test datasets (green) are much better approximated by our predicted curves (yellow) than they are by the traditional source test dataset curves (red). MIMIC-CXR was shortened to CXR in this figure.

Next we compared our estimated external accuracy, *P*_*Est*_, to the accuracy obtained on a matched external dataset, *P*_*Ext*_ (where available). Table 2 shows the source dataset model test set accuracy, estimated DABIS, the resultant estimated external accuracy, the obtained external dataset accuracy, the difference between source and external accuracies, the difference between estimated and external accuracy, and the accuracy obtained when running a model trained on shuffled source data on shuffled external data. Using the above technique, we also estimated the ROC curves in Fig. 2. For example, with the MIMIC-CXR dataset, we measure the AUROC of a DenseNet at 0.85. However, the MIMIC-CXR model only achieved 0.73 on the CXP dataset. This is a substantial drop in performance, indicating that the AI learned multiple shortcuts that failed to generalize outside of the original institution. Other dataset pairs yielded similar conclusions. In each case, there was a marked performance drop on the external dataset that is well estimated by equation (1). Specifically, there was a significant potential performance drop of 23% across multiple datasets and modalities. This 23% reduction in performance is calculated by subtracting our average *P*_DABIS_ estimate of 73% by 0.5, as any AUROC above random, i.e. 0.5, represents bias.

**Table 2:** Comparison of Source Data Model Performance, Estimated External Validation Performance, and Observed External Validation Performance on 13 Datasets and 5 Modalities

Source Dataset	*P*_Source_	*P*_DABIS_	*P*_Est_	Ext. Dataset	*P*_Ext_	Δ(*P*_Source_, *P*_Ext_)	Δ(*P*_Est_, *P*_Ext_)	Shuffled Ext.
CXR	0.85 [0.85-0.85]	0.63	0.73 [0.72-0.73]	CXP	0.73 [0.73-0.74]	0.12	**0.00**	0.50
CXR	0.85 [0.85-0.85]	0.63	0.73 [0.72-0.73]	NIH	0.76 [0.76-0.77]	0.09	**-0.03**	0.51
CXP	0.79 [0.79-0.79]	0.57	0.72 [0.72-0.73]	CXR	0.77 [0.77-0.77]	0.02	-0.05	0.45
CXP	0.79 [0.79-0.79]	0.57	0.72 [0.72-0.73]	NIH	0.76 [0.76-0.77]	0.03	-0.04	0.50
CXR+CXP	0.82 [0.82-0.82]	0.61	0.72 [0.71-0.72]	NIH	0.77 [0.77-0.78]	0.05	-0.05	0.51
CXR+NIH	0.85 [0.84-0.85]	0.68	0.67 [0.66-0.67]	CXP	0.69 [0.69-0.69]	0.16	**-0.02**	0.50
COVID-Ext	0.99 [0.98-0.99]	0.80	0.68 [0.67-0.69]	COVID-Int	0.64 [0.63-0.65]	0.36	**0.04**	0.53
ILD-Diag	0.95 [0.93-0.96]	0.85	0.60 [0.58-0.62]	ILD-Plan	0.66 [0.59-0.73]	0.29	**-0.06**	0.52
PTB-XL ECG	0.90 [0.89-0.91]	0.88	0.52 [0.52-0.53]	LUDB	0.70 [0.62-0.79]	0.21	**-0.18**	0.60
ICHBHI	0.97 [0.90-1.00]	0.91	0.57 [0.50-0.67]	JUST	0.60 [0.38-0.81]	0.37	**-0.03**	0.51
MIMIC-III	0.72 [0.68-0.76]	0.63	0.59 [0.54-0.63]	EHR-Int	0.58 [0.56-0.60]	0.14	**0.01**	0.51
**Average**:	0.87	0.73	0.64		0.68	0.20	-0.04	0.52

Results of AUROC model performance and bias estimates on validation and external datasets including 95% confidence intervals [A-B]. *P*_Source_, *P*_DABIS_, *P*_Est_, *P*_Ext_, are the source, DABIS, calibrated external estimate, and external AUROCs, respectively. Where a dataset appears in multiple rows, averages are calculated first across instances of the dataset, then across all datasets. Δ refers to the difference in AUROC between two estimates. Values in the Δ(*P*_Est_, *P*_Ext_) column that are bolded highlight instances where our DABIS estimate outperforms the Δ(*P*_Source_, *P*_Ext_) column. MIMIC-CXR was shortened to CXR in this Table. Est. and Ext. refer to estimated and external, respectively. Shuffled Ext. refers to results obtained by validating models trained on shuffled source datasets on shuffled external datasets. Reporting AI model accuracy without external validation overestimates model performance by 20%, whereas our method underestimates it by 4% on average.

We further evaluated a scenario where two datasets (MIMIC-CXR and CheXpert) were combined, observing a combined bias (*P*_DABIS_) of 0.61. Other clinical-ML studies have combined datasets from multiple hospitals, either with or without federated learning, to improve generalizability of ML models. To experiment with this we combined different pairs of datasets, (CXR and CXP), and (CXR and NIH). Our results demonstrate that combining two datasets does not mitigate bias. The CNN was instead able to learn and apply the distinct biases of each dataset, perhaps by first identifying the dataset the sample belongs to and then applying the corresponding per-hospital biased model shortcuts. For the (CXR and CXP) combination we used the NIH X-ray dataset as a third holdout set, and noted an observed drop in performance by 5% (0.82 to 0.77). By pairing MIMIC-CXR and NIH datasets, and using the CheXpert as the external dataset role we again observed a lack of generalizability. Interestingly the NIH dataset was consistently easier to generalize to than MIMIC-CXR or CheXpert, potentially owing to less complex cases.

Finally, we investigated our assumption that our shuffled data transform represents true shortcuts. We trained a model on shuffled data and tested that model on shuffled external validation data. The average shuffled model accuracy was 52% (Table 2), implying that 2% of our estimated DABIS did generalize to external datasets.

## Discussion

The findings of our study can be summarized as follows: data from 207,487 patients were collected across five modalities from 13 clinical datasets. Validation of models on external datasets determined that traditional training and evaluation methods overestimated accuracy on average by 20% due to hidden DABIS. In contrast, through estimation of DAB and model shortcuts our proposed method predicts external dataset model performance to within an average of 4% of measured performance across a multitude of datasets, tasks, and modalities (X-Rays, CTs, ECGs, clinical discharge summaries, and auscultation data). Additionally, we demonstrate that significant DAB exists in various large passively collected open-source medical datasets. For example, patients with presumed (but unconfirmed) interstitial lung disease may be biased toward specific or optimized imaging protocols that are intended to confirm the diagnosis, versus unsuspected cases that receive generic screening protocols. Taken together, our results shed light on a hidden driving factor for the lack of generalizability of AI models across a broad spectrum of unstructured clinical data by demonstrating the significant capacity of AI to learn misleading features from shortcuts.

To measure bias, we identified a heterogenous DABIS metric to estimate external dataset accuracy in unstructured data using one or more source datasets. We demonstrate that AI model results can be calibrated by measuring their bias to more reliably present model accuracy in publications with and without external data, and to improve the search for novel models that are less susceptible to DAB. While our algorithm does not completely capture all bias, our DABIS estimation method brings us an average of 16% closer than current methods, improving our understanding of accuracy and bias within medical AI models and datasets.

There are several limitations in this study that should be considered. Although focusing on DAB, it is also important to evaluate potentially discriminative biases within datasets as AI algorithms can potentially perpetuate inequalities and socioeconomic disparities^21^. Furthermore, future research is encouraged on other unstructured dataset modalities such as ultrasound, magnetic resonance imaging (MRI), positron emission tomography (PET) scans and wearables. With 866 samples our smallest source dataset is still reasonably large in the broader context of healthcare research. For smaller source datasets cross-validation could be used to better estimate *P*_*Source*_, and *P*_*DABIS*_, with *P*_*Est*_ calculated from the means across folds.

A related area of study is in detecting distribution shifts post-model deployment^22^. Distribution shifts, in particular covariate shifts, occur when there are notable alterations in the input features of the dataset between training and external deployment. DABs that change (either over time, or between hospitals) are both examples of covariate shifts. The shortcut features (DAB) shift, while the reproducible features (what we argue are more structural or semantic in nature) remain more fixed. There are other examples of covariate shifts, such as when a hospital changes from CT to MRI, that are not associated with DAB and our algorithm would not measure or anticipate them. There are already studies detecting and understanding distribution shifts in the literature post-model deployment. In contrast to other works, our model’s focus is designed as a pre-deployment method to predict how the model will respond to potential shifts in DAB. In deployment, our model should be paired with post-deployment methods that monitor for non-DAB changes, including label shifts.

While our model focuses on DAB, it is also important to note that demographic information such as age, race and gender can lead to underlying model biases that may influence the AI model to taking shortcuts^23^. However, we note that in addition to inter-hospital scanner variations, demographic biases could also be present as DABIS and thus are potentially accounted for in *P*_*Est*_. For example, sex impacts both disease prevalence and patient size and would thus be learnable to an extent by the shuffled models. Incorporating demographics directly into our DABIS estimate may improve its explainability in understanding the source of DAB, and is a direction for future work.

Our proposed shuffling bias transform assumes no prior knowledge of the bias in a dataset, and it is important to consider its limitations in shortcut detection. Anything that changes the histogram of the data for one disease class of patients more often than another disease class will be well detected. These could be visible or invisible biases to the human eye. Examples of invisible biases include scanner setting variations, whereas visible biases include a ruler or ECG leads on an X-ray. Each of these biases creates unique histogram features consistent across patients where they are prevalent. This also assumes that such biases in these examples have a greater influence on the histogram compared to diseases, otherwise the model might falsely mistake disease features for biases (see next paragraph). Our results strongly support that our assumptions hold for various unstructured datasets, diseases, and modalities. Although our model is capable of detecting invisible and visible biases that modify the data histogram, it does not measure them separately and therefore cannot differentiate between these two types of biases. Biases that are structural in nature, but do not change the histogram, would be undetected. For such datasets, other data transforms would be needed to obscure the human-intelligible signal while preserving the bias. Many additional transformers are possible with specialized knowledge of the task, like masking out the lungs in images for a pneumonia detection model. In such cases, algorithm 2 still holds by replacing our shuffling method with another transform and thus still provides a way to better estimate external accuracy.

Our accuracy estimation algorithm makes two key assumptions: that features suppressed by the bias transform will not generalize to external data, and the best effort is made to learn the bias with said model. The first assumption explains how our algorithm tends to underestimate model accuracy on external data in Table 2, some reproducible medical signal remains in the shuffled image that still generalizes to external data. Validating our shuffled models on shuffled external datasets shows that on average 2 % of the DAB is itself reproducible at multiple hospitals. However, it is debatable if such features are desirable or shortcuts. Obesity is a risk factor for cardiomegaly, but could also be distinctly observable in an X-ray histogram, and thus learnable in the shuffled image. If obesity were eliminated in a novel population, an AI model that relied upon it would lose accuracy, making highly accurate models without surrogates more desirable. For the second assumption, one could fail to appropriately tune hyperparameters and claim no bias exists. In these instances open science and data remain a powerful mitigation tool.

In contrast to current reporting procedures, our algorithm tends to underestimate model accuracy on external data. There are two potential causes: we slightly overestimate the DAB (as discussed above), or the model gravitates towards an unbiased feature instead (this could explain the 2% of our external accuracy estimation error not accounted for by DAB overestimation in the shuffled model column). Models are naturally more shortcut-resilient when the true signal is easier to detect than the DAB, e.g., a model to detect prosthetic metal hips on pelvic CT. However, it is important to specify that our method is not a strict lower bound of external performance. Model accuracy will be overestimated in the presence of DAB that is undetectable by the bias transform, but that still causes a shortcut, as may potentially be the case in MIMIC-III.

Generally, we observed that model accuracy was well correlated with its DABIS estimate (Spearman’s rank correlation 0.80), but not with the discrepancy between our estimated accuracy and the true external accuracy (Spearman’s rank correlation 0.24). Together these imply that models claiming higher accuracy may be primarily due to DABIS, but that our method is well positioned for low and high bias datasets alike.

While combining multiple datasets for model training and validation would ideally yield the best model, models can learn to identify the data source and fit to the associated DAB of that source, resulting in a substantial drop in performance upon external validation^3^. Federated learning is an emerging ML technique that enables privacy preservation and keeps data onsite while allowing the AI model exposure to a more diverse dataset^24^. A large federated COVID-19 study, validated data using data from 20 hospitals achieving an AUC of over 0.92 on their model with strong generalization to independent sites, demonstrating that DABIS can potentially be overcome with federated learning and sufficient data. However, federated learning would not help address systemic biases or those that are common amongst institutions. Although we encourage the exploration of federated learning techniques, we further recommend having completely held-out datasets from independent hospitals for validation and using our proposed approach to measure potential bias across the training institutions. Our data indicates that combining different datasets yields different generalizability, implying a nuanced relationship that needs further study.

Although there are no universally accepted laws in regard to ML practices, groups are starting to join to build guidelines. Point four of the ML guiding principles recently published by the U.S. FDA, Health Canada and the UK’s Medicines and Healthcare Products Regulatory Agency^25^ states that training and testing sets should be non-intersecting and that data and acquisition site factors should be considered. Our proposed method provides a means to specifically quantify how those factors may impact a model, which could greatly improve regulatory analysis.

In conclusion, we have provided key insights to AI model accuracy degradation on external datasets and are the first to develop simple yet powerful methods to estimate shortcut learning of data acquisition biases within unstructured datasets and calculate the expected accuracy of resultant models on external datasets. With our findings, methods, and recommendations we aim to both enable and promote the safer integration of AI models into clinical workflows. By providing a means to quantify this problem we also pave the way for research into new solutions in the form of new models or dataset processing methods that mitigate these biases.

## Methods

### Datasets

Thirteen datasets of unstructured data were used in this study including both previously published datasets and unpublished internal datasets. We trained our models on public datasets: MIMIC-CXR (Medical Information Mart for Intensive Care-Chest X-ray)^26^, MIMIC-III^27^, Stanford CheXpert Chest X-rays (CXP)^28^, COVID-Kaggle^29^, Physikalisch-Technische Bundesanstalt-XL ECG (PTB-XL)^30^, and the International Conference on Biomedical Health Informatics (ICBHI 2017) respiratory sound database^31^; and a novel Interstitial Lung Disease CT (ILD) dataset from two disjoint acquisition pathways (ILD-Diag, and ILD-Plan). We externally validated our models using data with the same modality from a different site including the National Institute of Health (NIH) X-ray dataset^32^, Lobachevsky University Electrocardiography Database (LUDB)^33^, the respiratory sound database from the Jordan University of Science and Technology Faculty of Computer and Information Technology & King Abdullah University Hospital (JUST)^34^, and novel COVID-19 X-ray, ILD, and EHR datasets from within our hospital network. Our models, sample size, training, and testing split are outlined in (Supplementary Table 1) and included in our online repository. Dataset details follow. For further information see original dataset publications.

#### Public datasets

**MIMIC-CXR**: MIMIC-CXR (Medical Information Mart for Intensive Care-Chest X-ray)is a set of 377,110 chest X-rays by Beth Israel Deaconess Medical Center collected between 2011 - 2016. The data was acquired from routine care over 5-years, with the same labels. From 14 disease classes and from which we select the 5 classes Cardiomegaly (64,346 X-rays), Edema (36,564 X-rays), Consolidation (14,675 X-rays), Atelectasis (65,047 X-rays), and Pleural Effusion (76,957 X-rays).

**MIMIC-III**: MIMIC-III is an electronic health record (EHR) database of de-identified health data from the Beth Israel Deaconess Medical Center. This dataset has over 40,000 patients and contains data on vital signs, laboratory measurements, medications, fluid balance, chart notes from healthcare providers, procedure codes, diagnostic codes, imaging reports, length of stay in the hospital, survival data, etc. The data was acquired from routine care over 11 years between 2001-2012. In this study, we focus on predicting 30-day readmission using clinical discharge summaries^19^. **Stanford CXP**: Stanford CheXpert Chest X-rays (CXP) is a recently released cohort of a set of 224,316 chest X-rays from Stanford Hospital. The data is acquired from routine care over 5-years, with a diverse set of labels (No Finding, Enlarged Cardiom., Cardiomegaly, Lung Lesion, Lung Opacity, Edema, Consolidation, Pneumonia, Atelectasis, Pneumothorax, Plural Effusion, Pleural Other, Fracture, and Support Devices). From this diverse set of labels we selected 5 classes Cardiomegaly (27,000 X-rays), Edema (52,246 X-rays), Consolidation (14,783 X-rays), Atelectasis (33,376 X-rays), and Pleural Effusion (86,187X-rays). **NIH**: The National Institute of Health (NIH)-14 dataset since its original publication is now a set of 112,120 frontal-view de-identified X-rays released in png format. It consists of labels from 14 disease classes and from which we select the 5 classes Cardiomegaly (2763 X-rays), Edema (2295 X-rays) Consolidation (4645 X-rays), Atelectasis (11,529 X-rays), and Pleural Effusion (13,276 X-rays) which are common with the MIMIC-CXR and the Stanford CXP dataset for evaluation. The original train-test splits released by the authors have been retained in our experiments too with no overlap occurring between the two sets. **COVID-19 Kaggle**: The COVID-19 Radiography Database from Kaggle is a database of chest X-rays images from 3616 COVID-19 positive images, 10,192 normal, 6012 lung opacity(non-COVID) and 1345 viral pneumonia images. For label alignment we used only COVID-19 positive and normal images. This dataset was curated from researchers at Qatar University, Doha, Qatar, and the University of Dhaka, Bangladesh and collaborators from Pakistan and Malaysia. **PTB-XL**: The Physikalisch-Technische Bundesanstalt XL(PTB-XL) ECG dataset is a large dataset of 21837 clinical 12-lead ECGs from 18885 patients of 10 second length recorded on Schiller AG devices over 7-years from 1989-1996 with labels of Normal ECG, Myocardial Infarction, ST/T Change, Conduction Disturbance, and Hypertrophy. From the 5 classes we utilized Conduction Disturbance (4907 ECGs) and Hypertrophy (2655 ECGs). **LUDB**: Lobachevsky University Electrocardiography Database (LUDB) database consists of 10-second 12-lead ECG signal records acquired on a Schiller Cardiovit AT-101 cardiograph. They collected ECGs from 200 patients with various cardiovascular diseases between 2017-2018 at the Nizhny Novgorod City Hospital No 5. To perform external validation on the PTB-XL model we filtered for patients that had cardiovascular diseases that fell into the “conduction disturbance” (66 ECGs) or “hypertrophy” (142 ECGs) classes which are classes also found in the PTB-XL ECG dataset. From the 200 Lobachevsky ECG dataset, 157 patients were selected for external validation for having an overlapping diagnosis with the PTB-XL ECG dataset. There were 91, 15, and 51 patients classified as hypertrophy, conduction disturbance and both hypertrophy an conduction disturbance, respectively. **ICBHI**: The International Conference on Biomedical Health Informatics (ICBHI) dataset is a publicly available audio samples dataset from the ICBHI 2017 Respiratory Challenge^31^ that we used as our training dataset. This has 5.5 hours of respiratory data from 126 patients with 6898 respiratory cycles, acquired from the Respiratory Research and Rehabilitation Laboratory (Lab3R). There were a total of 35 normal samples and 831 abnormal samples. **JUST**: The Jordan University of Science and Technology Faculty of Computer and Information Technology and King Abdullah University Hospital (JUST) dataset is a second set of audio data for evaluation comes from the King Abdullah University Hospital in Jordan. This can be considered as equivalent to the field acquired data, with data from 112 patients being captured via a Bluetooth attached electronic stethoscope^34^. These have been annotated for pulmonary lung conditions. There were a total of 105 normal samples and 138 abnormal samples. For label alignment between the two sets of data, we reduce the labeling to normal vs abnormal.

#### Internal datasets

All internal datasets were collected with institutional approvals and consent was waived due to the retrospective nature of the study, and was determined to be minimal risk to patients. **ILD-Diag**: Interstitial Lung Disease (ILD) diagnostic CT image volumes were collected from the Joint Department of Medical Imaging (JDMI) including Sinai Health System, Toronto Western Hospital, Princess Margaret Cancer Centre, Toronto General Hospital, and Women’s College Hospital. Diagnostic CT images were acquired between 2010 and 2018. Image volumes are predominately 3 mm slice-thickness, but where that was unavailable the image volume with slice-thickness nearest 3 mm was taken. In total there were 3028 samples without ILD and 1366 abnormal samples with ILD gathered for AI training. All 1366 abnormal samples with ILD were double-read by a cardiothoracic radiologist with 15 years of experience for confirmation. **ILD-Plan**: For external accuracy assessment of the ILD model radiotherapy CT simulation images were acquired between 2004 and 2015 for all patients undergoing stereotactic body radiation therapy planning for lung cancer at Princess Margaret Cancer Centre. These radiotherapy simulation images were acquired on independent scanners in a separate hospital department, with distinct imaging protocols and hardware (e.g. 2 mm slices, respiratory-correlated image reconstruction, modified patient positioning). There were no other exclusion criteria. For this external accuracy dataset, there were 448 samples without ILD and 55 abnormal samples. **COVID-Int**: Chest X-ray images acquired at the University Health Network from the beginning of the COVID-19 pandemic in March 2020 thru to November 2020. All images were included for which a patient had a COVID-19 polymerase chain reaction (PCR) test. Samples were determined positive for COVID-19 based on ground truth by PCR tests. Of the images acquired, 5131 were acquired on a fixed chest X-ray and 5600 were acquired on a portable chest X-ray. There were a total of 3409 COVID positive X-rays and 7322 normal X-rays. **EHR-Int**: Electronic health record (EHR) data that includes raw text of clinical discharge summaries of patients was collected during January 2020 to August 2020 from University Health Network hospitals (Toronto Western Hospital, Princess Margaret Cancer Centre, and Toronto General Hospital). There were 6278 no readmission samples and 986 readmission samples.

### Data processing

**Chest-X-rays**: For the X-ray datasets MIMIC-CXR,CXP,NIH, the samples were downsized to 320 × 320 pixels for input into the CNN. During the data download, a few files were corrupted and these were removed during the data processing stage (1 from CXP and 15 from MIMIC-CXR datasets). **ECG**: All 12-lead ECG data were in the lead order of [’i’, ’ii’, ’iii’, ’avr’, ’avl’, ’avf’, ’v1’, ’v2’, ’v3’, ’v4’, ’v5’, ’v6’], with a sampling rate of 1000. **COVID-X-rays**: The COVID-X-rays were obtained from two sources. The external (public) set was obtained from Kaggle^35^ and were made available in png format. The samples for the internal dataset were obtained as DICOM files and the array representation was used. The samples were resized to 256 × 256 and fed into the CNN. **ILD**: The data samples were resampled to 256 × 256x128 (height x width x depth) voxels from corresponding digital imaging and communications in medicine (DICOM) data files before being fed into the CNN. **Auscultation Data**: The audio samples were trimmed to 5 seconds and converted into mel-spectrogram images. This was then resized to 224 × 224 to be fed into the CNN. For label harmonization between the ICBHI and JUST datasets, normal/healthy labeled data was considered as “Normal" and samples with labels asthma, COPD, Pneumonia were considered as “Abnormal" and this was reduced to a 2 class classification problem. **EHR**: Clinical discharge summaries required the following pre-processing steps: conversion of words into lowercase, removal of line breaks and carriage returns, de-identification of personally identifiable info found inside brackets, removal of special characters such as “=,–", and usage of the SpaCy^36^ sentence segmentation package. The text was split into subsequences of 318 words and tokenized using BERT^18^ tokenizer. All data, except for the discharge summary data, was normalized based on mean and standard deviation based on population (ECGs were normalized per lead) prior to input into the AI models.

### Model architecture and training parameters

**X-ray models**: To evaluate DABIS in chest x-rays we used a DenseNet-121 CNN model for classification of disease targets. We used Adam optimizer with a learning rate set to 1*e* − 4 and beta values set to (0.9, 0.999). The models were trained for 10 epochs with learning rates being reduced by a factor of 10 in case of no AUC improvement on the validation set every $\lfloor \frac{E}{3}\rfloor$ epochs where E is the number of epochs. For smaller datasets where convergence was not achieved in 10 epochs ie. there was improvement to AUC on the validation set on the 10th epoch, the training was allowed to continue training for 50 epochs. **ECG model**: To evaluate DABIS in ECG, a simple five layer convolutional neural network model that was based on VGG-16^17^ architecture was used for classification of disease targets. Training was performed for 50 epochs with the same optimizer settings as that of the X-Ray model training. **CT model**: For the ILD Dataset, a 3D variant of the VGG network was used with each 2D convolution layer replaced with a 3D convolution. It consistent of 14 layers including a fully connected layer. The configuration for the layers is as [4, ’M’, 8, ’M’, 16, 32, ’M’, 32,32, ’M’, 64, 64, ’M’], with ’M’ representing a 3D maxpooling layer and the numbers representing the number of filters in each convolution layer. **Auscultation model**: A ResNet-34 model was used to classify the lung sounds data. The final fully connected layer had two output nodes for the normal and abnormal classes respectively. **EHR models**: To evaluate DABIS in electronic health record data, we used the ClinicalBERT large language model for sequence classification on BERT tokenized text from clinical discharge summaries^19^. ClinicalBERT is a Bidirectional Encoder Representations from Transformers model^18^ fine-tuned for medical text classification tasks.

#### Algorithm 1

Data Shuffling

**Input:** Data Tensor *D* of dimension *B* × *C* × *H* × *W*

**Output:** Shuffled Data Tensor *D* of dimension *B* × *C* × *H* × *W*

1: **for** sample *s* in batch *B* **do**

2: *permutation*_*idx* ← Random Permutation(*H*, *W*)

3: **for** channel *ch* in channels *C* **do**

4: Randomly permute in-place *D*[*s*, *ch*, *permutation*_*idx*]

5: **end for**

6: **end for**

7: **return** *D*

#### Algorithm 2

Estimate External Accuracy

1: **function** TRAIN_AND_TEST (*model*, *dataloaders*)

2: Define loss function and optimizer

3: Initialize early stopping counter to zero

4: Initialize best AUC to zero

5: Train the model:

6: **for** each epoch **do**

7: **for** each batch in the train dataloader **do**

8: Make a forward pass through the model

9: Compute the loss

10: Backpropagate the gradients and update the weights

11: **end for**

12: Evaluate the model on the validation dataloader

13: Compute the AUC on the validation set

14: **if** the AUC is better than the current best AUC **then**

15: Update the best AUC

16: Save the model weights

17: Reset the early stopping counter to zero

18: **else**

19: Increment the early stopping counter by 1

20: **end if**

21: **if** the early stopping counter reaches a pre-defined maximum **then**

22: Stop training and return the best model weights

23: **end if**

24: **end for**

25: Evaluate the model on the test dataloader

26: Compute the AUC on the test set

27: **return** AUC on the test set

28: **end function**

29: **function** ESTIMATE_EXTERNAL_ACC (*SourceData*)

30: Create non-overlapping train, validation, and test sets

31: Create train, validation, and test dataloaders

32: Define model and initialize parameters

33: *P*_*Source*_ ← TRAIN_AND_TEST (*model*, *dataloaders*)

34: Reset model parameters

35: Update dataloaders to apply Data Shuffling on each batch

36: *P*_*DABIS*_ ← TRAIN_AND_TEST (*model*, *dataloaders*)

37: Set $\bar{O}\leftarrow 0.5$                    ⊳(performance of a purely random model, .5 since it is AUROC)

38: $${P}_{Est}\leftarrow {P}_{Source}-{P}_{DABIS}+\bar{O}$$

39: **return***P*_*Est*_

40: **end function**

### Model performance

Performance for multi-class models were evaluated using Area Under the Receiver Operating Characteristic Curve (AUROC) in a “One vs All” method. The AUROC can be understood as the probability of correctly classifying a random pair of samples as class one versus two. We calculated the confidence intervals using 1000 bootstraps and the data resampling function from roc-utils^37^.

### Reporting summary

Further information on research design is available in the Nature Research Reporting Summary linked to this article.

### Supplementary information

Reporting Summary

Supplementary Table

## Data Availability

The MIMIC-CXR, MIMIC-III, CXP, NIH, COVID-Kaggle, PTB-XL ECG, LUDB, ICBHI and JUST datasets are all publicly available. Requests for the raw images and associated Digital Imaging and Communications in Medicine data in the ILD, COVID, and EHR Internal datasets should be directed to C.M.

## Code availability

Analysis code for building dataset splits, training models, estimating bias, and creating estimated ROC curves is available at https://github.com/mcintoshML/Data-Bias-Analysis.
