# Supplementary material for: Shortcut learning in medical AI hinders generalization: method for estimating AI model generalization without external data
Source: NPJ Digit Med. 2024 May 14;7:124. doi: 10.1038/s41746-024-01118-4 (PMC11094145; doi:10.1038/s41746-024-01118-4)
Supplement: Supplementary file 1 — Reporting Summary [file 41746_2024_1118_MOESM1_ESM.pdf]

nature portfolio

Corresponding author(s): Chris McIntosh

Last updated by author(s): Apr 2, 2024

Reporting Summary

Nature Portfolio wishes to improve the reproducibility of the work that we publish. This form provides structure for consistency and transparency in reporting. For further information on Nature Portfolio policies, see our Editorial Policies and the Editorial Policy Checklist.

Statistics

For all statistical analyses, confirm that the following items are present in the figure legend, table legend, main text, or Methods section.

n/a	Confirmed	
☐	☒	The exact sample size (n) for each experimental group/condition, given as a discrete number and unit of measurement
☐	☒	A statement on whether measurements were taken from distinct samples or whether the same sample was measured repeatedly
☒	☐	The statistical test(s) used AND whether they are one- or two-sided<br>*Only common tests should be described solely by name; describe more complex techniques in the Methods section.*
☒	☐	A description of all covariates tested
☒	☐	A description of any assumptions or corrections, such as tests of normality and adjustment for multiple comparisons
☐	☒	A full description of the statistical parameters including central tendency (e.g. means) or other basic estimates (e.g. regression coefficient) AND variation (e.g. standard deviation) or associated estimates of uncertainty (e.g. confidence intervals)
☒	☐	For null hypothesis testing, the test statistic (e.g. F, t, r) with confidence intervals, effect sizes, degrees of freedom and P value noted<br>*Give P values as exact values whenever suitable.*
☒	☐	For Bayesian analysis, information on the choice of priors and Markov chain Monte Carlo settings
☒	☐	For hierarchical and complex designs, identification of the appropriate level for tests and full reporting of outcomes
☒	☐	Estimates of effect sizes (e.g. Cohen's d, Pearson's r), indicating how they were calculated

Our web collection on statistics for biologists contains articles on many of the points above.

Software and code

Policy information about availability of computer code

Data collection	External datasets were downloaded from:<br>MIMIC-CXR: https://physionet.org/content/mimic-cxr/2.0.0/<br>MIMIC-III: https://physionet.org/content/mimiciii/1.4/<br>Stanford CXR: https://stanfordmlgroup.github.io/competitions/chexpert/<br>NIH: https://nihcc.app.box.com/v/ChestXray-NIHCC<br>PTB-XL: https://physionet.org/content/ptb-xl/1.0.1/<br>LUDB: https://physionet.org/content/ludb/1.0.1/<br>ICBHI: https://bhichallenge.med.auth.gr/ICBHI_2017_Challenge<br>JUST: https://data.mendeley.com/datasets/jwyy9np4gv/3<br><br>Internal dataset was collected from DICOM using our clinical PACS.<br>DICOMs were loaded using pydicom (ver 2.2.2) and analyzed using custom written Python code (ver 3.8.11).
Data analysis	Data analysis was performed in Python (ver 3.8.11)<br>Code available on github: https://github.com/mcintoshML/Data-Bias-Analysis

For manuscripts utilizing custom algorithms or software that are central to the research but not yet described in published literature, software must be made available to editors and reviewers. We strongly encourage code deposition in a community repository (e.g. GitHub). See the Nature Portfolio guidelines for submitting code & software for further information.

Data

Policy information about availability of data

All manuscripts must include a data availability statement. This statement should provide the following information, where applicable:

- Accession codes, unique identifiers, or web links for publicly available datasets
- A description of any restrictions on data availability
- For clinical datasets or third party data, please ensure that the statement adheres to our policy

The MIMIC-CXR, MIMIC-III, CXP, NIH, COVID-Kaggle, PTB-XL ECG, LUDB, ICBHI and JUST datasets are all publicly available. Requests for the raw images and associated Digital Imaging and Communications in Medicine data in the ILD, COVID-Internal, and EHR-Internal datasets should be directed to C.M.

Research involving human participants, their data, or biological material

Policy information about studies with human participants or human data. See also policy information about sex, gender (identity/presentation), and sexual orientation and race, ethnicity and racism.

Reporting on sex and gender	Sex and gender are available from original authors of datasets.<br>Gender is not available for analysis on internal datasets
Reporting on race, ethnicity, or other socially relevant groupings	Disclosed by original authors for public datasets. Race, ethnicity, or other socially relevant groupings information not available for internal datasets.
Population characteristics	Disclosed by original authors for public datasets. Population characteristic information not available for internal datasets.
Recruitment	Data was gathered through routine clinical care. This study investigates data acquisition bias resulting from routine clinical care and AI.
Ethics oversight	Public datasets were obtained and used under their guidelines/permissions. Internal datasets were collected retrospectively with approval of institutional review boards.

Note that full information on the approval of the study protocol must also be provided in the manuscript.

Field-specific reporting

Please select the one below that is the best fit for your research. If you are not sure, read the appropriate sections before making your selection.

☒ Life sciences ☐ Behavioural & social sciences ☐ Ecological, evolutionary & environmental sciences

For a reference copy of the document with all sections, see nature.com/documents/nr-reporting-summary-flat.pdf

Life sciences study design

All studies must disclose on these points even when the disclosure is negative.

Sample size	No sample size calculation was performed. Sample size was determined based on availability of data. Training and validation sample sizes were determined by a random 80/20 split. Full datasets were used for external validation.
Data exclusions	For details on external datasets see original publications. For the COVID dataset all data with patients greater than the age of 17 with a polymerase chain reaction test result available were collected. For the ILD diagnostic dataset there were no exclusion criteria. For the ILD planning data all patients undergoing stereotactic body radiation therapy planning were included.
Replication	All studies were validated against separate external datasets. Except for ILD dataset which was a held-out distinct dataset (wording in paper) acquired in different area of hospital. To calculate AUC 1000 bootstraps were performed. Code available on Github link reported under code availability in manuscript.
Randomization	Samples were randomized into training and validation splits. To eliminate bias from biological signal we shuffled the signal to determine signal bias.
Blinding	Data was acquired by clinician during routine clinical care, retrospectively gathered. Blinding of the authors was therefore not necessary.

Reporting for specific materials, systems and methods

We require information from authors about some types of materials, experimental systems and methods used in many studies. Here, indicate whether each material, system or method listed is relevant to your study. If you are not sure if a list item applies to your research, read the appropriate section before selecting a response.

Materials & experimental systems

n/a	Involved in the study
☒	☐ Antibodies
☒	☐ Eukaryotic cell lines
☒	☐ Palaeontology and archaeology
☒	☐ Animals and other organisms
☐	☒ Clinical data
☒	☐ Dual use research of concern
☒	☐ Plants

Methods

n/a	Involved in the study
☒	☐ ChIP-seq
☒	☐ Flow cytometry
☒	☐ MRI-based neuroimaging

Clinical data

Policy information about clinical studies
All manuscripts should comply with the ICMJE guidelines for publication of clinical research and a completed CONSORT checklist must be included with all submissions.

Clinical trial registration	N/A
Study protocol	N/A
Data collection	See above.
Outcomes	N/A
