# Supplementary material for: Shortcut learning in medical AI hinders generalization: method for estimating AI model generalization without external data
Source: NPJ Digit Med. 2024 May 14;7:124. doi: 10.1038/s41746-024-01118-4 (PMC11094145; doi:10.1038/s41746-024-01118-4)
Supplement: Supplementary file 2 — Supplementary Table [file 41746_2024_1118_MOESM2_ESM.pdf]

Supplemental Material – Shortcut Learning in Medical AI Hinders Generalization: Method for Estimating AI Model Generalization without External Data

Supplementary Table 1. Class distribution of datasets. The label counts corresponding to each class in the dataset is indicated in the tables

Class	Dataset		
	CXP	MIMIC-CXR	NIH
No Finding	22381	143352	60184
Enlarged Cardiomediastinum	10798	10042	0
**Cardiomegaly**	27000	64346	2763
Lung Opacity	105581	76423	0
Lung Lesion	9186	10801	0
**Edema**	52246	36564	2295
**Consolidation**	14783	14675	4645
Pneumonia	6039	26222	1428
**Atelectasis**	33376	65047	11529
Pneumothorax	19448	14257	5289
**Pleural Effusion**	86187	76957	13276
Pleural Other	3523	3460	0
Fracture	9040	7605	0
Support Devices	116001	84073	0

(a) X-ray diagnostics data

Class	Dataset	
	COVID-Kaggle	COVID-Internal
**COVID**	3616	3409
**Normal**	10192	7322
Lung Opacity	6012	0
Viral Pneumonia	1345	0

(b) COVID-19 data

Class	Dataset	
	ILD-Diag	ILD-Plan
**Abnormal**	1366	55
**Normal**	3028	448

(c) CT data

Class	Dataset	
	JUST	ICBHI
**Abnormal**	138	831
**Normal**	105	35

(d) Auscultation data

Class	Dataset	
	PTB-XL ECG	LUDB
**CD**	4907	66
**HYP**	2655	142
MI	5486	
STTC	5250	
NORM	9528	

(e) ECG data

Class	Dataset	
	MIMIC-III	EHR-Int
**No Readmission**	15901	6278
**Readmission**	16444	986

(f) Discharge summary data
